# Association Between TSH Level and Stroke Severity: A Cross‐Sectional Study Using the mRS

**DOI:** 10.1155/srat/2316652

**Published:** 2025-12-18

**Authors:** Afshin Moradi, Asal Ebrahimian, Seyed Ardalan Alhoseini, Kosar Baghernezhad, Amir Ghaffarzad, Sona Abolhasani, Vahideh Sadra, Samad Shams Vahdati

**Affiliations:** ^1^ Student Research Committee, Tabriz University of Medical Sciences, Tabriz, Iran, tbzmed.ac.ir; ^2^ Emergency and Trauma Care Research Center, Imam Reza General Hospital, Tabriz University of Medical Sciences, Tabriz, Iran, tbzmed.ac.ir; ^3^ Neurosciences Research Center (NSRC), Tabriz University of Medical Sciences, Tabriz, Iran, tbzmed.ac.ir; ^4^ Endocrine Research Center, Imam Reza General Hospital, Tabriz University of Medical Sciences, Tabriz, Iran, tbzmed.ac.ir

**Keywords:** cross-sectional, mRS, stroke, thyroid-stimulating hormone, TSH

## Abstract

**Background:**

Stroke is a major global health issue with numerous contributing risk factors. One of the most important risk factors of stroke is cardiovascular disease. Other risk factors include dyslipidemia, obesity, diabetes, and thyroid dysfunction which are known to increase the risk of cardiovascular disease. As dysfunction of the thyroid and its related hormones such as thyroid‐stimulating hormone (TSH) are in correlation with cardiovascular disease, which may lead to stroke, investigating the correlation between TSH levels and the severity of stroke is worth attention.

**Methods:**

This cross‐sectional study was conducted in Imam Reza Hospital in Tabriz, Iran, from March 2021 to March 2023. In this study, 204 stroke patients whose TSH levels were evaluated were included. Data on age, gender, comorbidities, TSH levels, stroke subtype, and disability severity (measured by the modified Rankin scale [mRS] before admission, during admission, at discharge, and after 3 months) were analyzed using SPSS Version 22.

**Results:**

Out of 204 patients, TSH levels of 35 (17.2%) patients were below the range (TSH < 0.39), 154 (75.5%) patients within the range (TSH: 0.39–6.19), and 15 (7.4%) patients had TSH levels above the range (TSH > 6.19). There is a weak to moderate but statistically significant correlation between mRS and TSH level during admission (*R* = −0.25, *p* < 0.01) and at discharge (*R* = −0.28, *p* < 0.01). However, there is a nonsignificant correlation before admission (*R* = −0.10, *p* = 0.174) and 3 months after discharge (*R* = −0.12, *p* = 0.137). There is not a significant difference among TSH groups comparing their age, gender, type of stroke, and CT scan findings.

**Conclusion:**

Elevated TSH levels may be associated with less severe stroke symptoms at both admission and discharge. Further studies with larger sample sizes are necessary to better understand the relationship between thyroid function and stroke.

## 1. Introduction

Stroke is a neurological deficit and one of the leading causes of disability and death in older adults [1]. It is broadly classified into three major pathological subtypes: ischemic stroke, intracerebral hemorrhage, and subarachnoid hemorrhage [2, 3]. The overall stroke prevalence was 2.5% in the United States, and the incidence of stroke in Iran was 102,778 patients in 2019 [4, 5]. There are various scoring systems for classifying the performance of stroke patients; one of which is called the modified Rankin scale (mRS). The mRS score is the most common tool that has been used by clinicians and researchers to quantify stroke disability [6, 7]. The mRS score is divided into seven categories, taking into account the level of activity, physical performance, and patient participation. A score of 0 means complete health and no symptoms while a score of 6 represents death (Table 1) [8].

**Table 1:** mRS scoring system.

**Grade**	**Symptoms**
0	None
1	No significant disability despite symptoms: Able to carry out all usual duties and activities
2	Slight disability: Unable to carry out all previous activities, but able to look after own affairs without assistance
3	Moderate disability: Requiring some help, but able to walk without assistance
4	Moderately severe disability: Unable to walk without assistance, unable to attend to needs without assistance
5	Severe disability: Bed‐ridden, incontinent, and requiring constant nursing care and attention
6	Dead

There are many risk factors for stroke; one of which is cardiovascular diseases that are reported to have a huge impact on stroke occurrence [9]. Additionally, many factors such as dyslipidemia, obesity, diabetes, and thyroid dysfunction (hypothyroid, hyperthyroid) are known to be associated with increasing cardiovascular disorders which may later be followed by ischemic stroke. As thyroid dysfunctions are mentioned to be an important risk factor for stroke, investigating the correlation between thyroid hormones (T3, T4) and TSH levels and severity of stroke warrants attention [10–12]. Several recent studies have addressed the impact of diabetes management, anticoagulation control, and neuroprotective agents on stroke outcomes [13–15]. Therefore, in this study, we aimed to evaluate TSH levels among patients with different stroke subtypes and explore their correlation with stroke severity using the mRS.

## 2. Methods

### 2.1. Study Design

This cross‐sectional study followed the STROBE (Strengthening the Reporting of Observational Studies in Epidemiology) guidelines, which are widely recommended to ensure the transparent and standardized reporting of observational research.

### 2.2. Setting

All patients who were admitted to Imam Reza Hospital of Tabriz, affiliated to Tabriz University of Medical Sciences, Tabriz, Iran, and were hospitalized with a definite stroke diagnosis from March 2021 to March 2023 were included in the study.

### 2.3. Participants

#### 2.3.1. Inclusion Criteria

Patients diagnosed with stroke who had documented thyroid function test results during their hospital admission were included.

#### 2.3.2. Exclusion Criteria

Patients with a history of using any medications like dobutamine, glucocorticoids, bromocriptine, dopamine, and other substances that can affect TSH levels were excluded.

### 2.4. Variables

Data were collected on patient demographics (age, gender), comorbidities, TSH levels (mIU/L), stroke subtype, and stroke severity. Stroke severity was assessed using the mRS at discharge and 3 months postdischarge.

### 2.5. Measurements

TSH levels of stroke patients were measured via blood sample collection at the time of admission, and the severity of stroke was assessed by using the mRS by a trained neurologist.

### 2.6. Statistical Methods

For statistical data analysis, SPSS Version 22 was used. The data were reported descriptively (frequency and percentage), with mean ± standard deviation (SD) or median and interquartile range (IQR). To assess the normality of the data, the Kolmogorov–Smirnov test was used. For quantitative variables with normal distribution, the independent sample *t*‐test was applied; for nonnormally distributed quantitative data, the Mann–Whitney *U* test was used; and for qualitative data, the chi‐square test was performed. In this study, a *p* value of less than 0.05 was considered statistically significant and 95% confidence intervals were calculated for all statistical estimates.

## 3. Results

In this study, 204 patients with a stroke diagnosis and available thyroid tests were included. One hundred nine (109, 53.4%) patients were female. The median age of participants was 66 years (IQR: 23). The youngest patient was 18 years old, and the oldest was 95 years old. Out of 204 stroke patients, 151 (74%) patients were diagnosed with ischemic stroke, 33 (16.2%) patients were diagnosed with hemorrhagic stroke, 11 (5.4%) patients had cerebral venous thrombosis (CVT), and 9 (4.4%) patients had transient ischemic attack (TIA). Demographic and clinical information of patients is presented in Table 2.

**Table 2:** Demographic and clinical information of patients.

**Baseline characteristic**	**Patients**	
	**n**	**%**
Gender		
Female	109	53.4%
Male	95	46.6%
Type of stroke		
Ischemic	151	74%
Hemorrhagic	33	16.2%
Transient ischemic attack (TIA)	9	4.4%
Cerebral venous thrombosis (CVT)	11	5.4%
Pre‐existing conditions		
Hypertension	129	63.2%
Diabetes	67	32.8%
Ischemic heart disease	26	12.7%
History of open‐heart surgery	7	3.4%
Valvular heart disease	2	1%
History of heart valve surgery	4	2%
Atrial fibrillation	14	6.9%
Heart failure	6	2.9%
History of myocardial infarction (heart attack)	2	1%
History of stroke	49	24%
History of deep vein thrombosis (DVT)	5	2.5%
History of transient ischemic attack (TIA)	6	2.9%
History of cerebral venous thrombosis (CVT)	3	1.5%
History of oral contraceptive pill (OCP) use	5	2.5%
Hyperlipidemia	21	10.3%

Among 204 patients for whom TSH levels were measured, the median measured TSH level was 1.3, with an IQR of 2.05. For stroke patients, 35 (17.2%) patients had a level of TSH below the range (TSH < 0.39), 154 (75.5%) patients had TSH levels within the range (TSH: 0.39–6.19), and 15 (7.4%) patients had TSH levels above the range (TSH > 6.19). The level of TSH in stroke patients is shown in Table 3.

**Table 3:** The level of TSH in patients with stroke and their categorization.

**Characteristic**	**Patients**	
	**n**	**%**
TSH < 0.39	35	17.2%
TSH: 0.39–6.19	154	75.5%
TSH > 6.19	15	7.4%

The relationship between the severity of stroke based on mRS and TSH levels was evaluated. Based on standard interpretation thresholds, correlations were categorized as weak, moderate, or strong. The results showed that higher TSH levels were weakly associated with lower disability scores during admission and at discharge, but not before or 3 months after hospitalization. Regarding associations between other variables and TSH level, we found that male gender had a statistically significant negative correlation with the level of TSH (Table 4).

**Table 4:** The relationship between the variables and severity of stroke based on mRS and TSH levels.

	**R**	**p** ** value**
mRS before admission	−0.10	0.174
mRS during admission	−0.25 ^∗^	< 0.01
mRS at discharge	−0.28 ^∗^	< 0.01
mRS 3 months after discharge	−0.12	0.137
Age	−0.092	0.190
Male gender	−0.193 ^∗^	0.006
Hypertension	−0.010	0.891
Diabetes	0.066	0.351
Ischemic heart disease	−0.008	0.908
History of open‐heart surgery	−0.012	0.868
Valvular heart disease	−0.013	0.857
History of heart valve surgery	−0.032	0.651
Atrial fibrillation	−0.007	0.920
Heart failure	0.034	0.632
History of myocardial infarction (heart attack)	0.130	0.063
History of stroke	−0.013	0.849
History of deep vein thrombosis (DVT)	0.058	0.413
History of transient ischemic attack (TIA)	0.051	0.741
History of cerebral venous thrombosis (CVT)	−0.003	0.965
History of oral contraceptive pill (OCP) use	0.029	0.680
Hyperlipidemia	−0.021	0.764

^∗^Statistically significant.

The analysis of various comparative characteristics and outcomes of stroke patients which were stratified by TSH levels into three groups (TSH < 0.39, TSH 0.39–6.19, and TSH > 6.19) showed that age, gender, type of stroke, and CT scan findings are not significantly different among the TSH groups. However, significant differences in mRS scores during admission and discharge were indicated (Table 5).

**Table 5:** Differences in age, gender, mRS score, stroke type, and CT scan findings among TSH groups.

	**T** **S** **H** < 0.39	**TSH: 0.39–6.19**	**T** **S** **H** > 6.19	**p** ** value**
Age (median [IQR])	67 (28)	63 (26)	55.5 (32)	0.63
Gender				
Female	20 (57.1%)	78 (50.6%)	11 (73.3%)	0.22
Male	15 (42.9%)	76 (49.4%)	4 (26.7%)	
mRS before admission (median [IQR])	0 (1)	0 (1)	0 (0)	0.62
mRS during admission (median [IQR])	4 (2)	3 (2)	3 (2)	0.03
mRS at discharge (median [IQR])	4 (3)	3 (3)	3 (3)	0.03
mRS 3 months after discharge (median [IQR])	3 (4)	3 (3)	3 (4)	0.74
Type of stroke				
TIA	1 (2.9%)	8 (5.2%)	0 (0%)	0.85
Ischemic	25 (71.4%)	114 (74.0%)	12 (80.0%)	
Hemorrhagic	7 (20.0%)	23 (14.9%)	3 (20.0%)	
CVT	2 (5.7%)	9 (5.8%)	0 (0%)	
CT scan findings				
Nonhemorrhagic changes	8 (22.9%)	27 (17.5%)	4 (26.7)	0.56
Cerebral hemorrhagic	7 (20.0%)	20 (13.0%)	3 (20.0%)	
Normal	20 (57.1%)	107 (69.5%)	8 (53.3%)	

## 4. Discussion

Stroke is an acute neurological event that acts as a physiological stressor, negatively impacting various neurophysiological pathways [16]. Thyroid hormone dysfunction is a possible risk factor for stroke. In a cross‐sectional study by Al‐Mahdawi et al., which was conducted among 105 patients with ischemic stroke, it was reported that hypothyroidism has been strongly associated with the occurrence of ischemic stroke; the mechanism behind which is explained by the decrease in heart rate and the increase in vascular resistance and blood pressure that follow hypothyroidism [17]. Another study by Chaker et al. indicated that a higher level of TSH decreases the risk of stroke. The study concluded that there is a 1.28‐fold increase in the risk of stroke in individuals whose TSH levels are in the lower range of normal compared to those whose TSH levels are in the upper range of normal [18].

A retrospective cohort study by Papaleontiou et al. found a 3.8‐fold increase in the occurrence of atrial fibrillation (AF) and a 4.2‐fold increase in stroke incidence over a 5‐year follow‐up in individuals with TSH < 0.1 mIU/L. Additionally, a 1.8‐fold increase in AF risk and a 3.6‐fold increase in stroke incidence were observed in individuals with TSH > 5.5 mIU/L during the same period, with similar results seen in older age groups. Therefore, it was found that both hyperthyroidism and hypothyroidism are associated with an increased risk of stroke [19].

A 5‐year cohort study by Sheu et al. showed that both hyperthyroidism and antithyroid treatment were associated with short‐term and long‐term cerebrovascular complications and an increased risk for ischemic stroke among young adults. They reported that the hazard of having ischemic stroke during the 5‐year follow‐up period was 1.44 times greater (*p* = 0.038) for patients with hyperthyroidism [20].

A study conducted by Pande et al. included 185 patients diagnosed with hemorrhagic stroke and reported the prevalence of hyperthyroidism and hypothyroidism to be 8.1% and 5.9%, respectively. They observed that low FT3 and low FT4 predict a poor outcome in patients with hemorrhagic stroke.

Another study by Höfler et al. included 107 patients diagnosed with CVT. Results showed that thyroid dysfunction was present in 17.8% of patients as 15.9% of them had hypothyroidism and 1.9% of them had hyperthyroidism. They concluded that thyroid dysfunction was remarkably and highly prevalent among CVT patients [21, 22].

Our study showed that TSH levels were not significantly associated with stroke occurrence or 3‐month prognosis. However, TSH levels were significantly associated with the severity of symptoms at the time of admission and discharge as increased TSH levels were, along with lower severity of symptoms. For dealing with possible confounding factors that may affect this association, multivariate analysis was conducted and results did not show statistically significant confounders. Notably, although gender was found to be associated with TSH levels, it did not act as a confounder in the association between TSH and stroke severity. Similar to our findings, a study by Delpont et al. has investigated the association between TSH level and the severity of ischemic stroke symptoms. They enrolled 731 ischemic stroke patients and measured serum TSH levels at admission. Among patients, 629 (86%) had TSH levels in the normal range (0.36–3.74 mUI/L), 64 (8.6%) had low TSH levels (TSH < 0.36 mUI/L), and 38 (5.1%) had high TSH levels (TSH > 3.74 mUI/L). For assessing the severity of stroke at admission and discharge, the National Institutes of Health Stroke Scale (NIHSS) and mRS were used, respectively. TSH levels were categorized into tertiles based on their distribution (< 0.822, 0.822–1.6, and > 1.6 mUI/L) due to the violation of the normality assumption. Results showed that a higher level of TSH at admission was significantly and independently associated with a lower severity score both at admission and better functional outcome at discharge in patients with acute ischemic stroke [23]. In a systematic review and meta‐analysis conducted by Dhital et al., studies investigating the association between thyroid function and functional stroke outcomes were included. The analysis of a total of 5218 patients reported that subclinical hypothyroidism was associated with significantly better functional outcomes at 1 and 3 months after stroke as measured by mRS (OR 2.58 and OR 2.28, respectively). Additionally, higher baseline TSH and T3 were predictive of favorable recovery suggesting the potential neuroprotective role of thyroid hormones in stroke rehabilitation. They concluded that elevated initial serum TSH levels were associated with a lower severity score at admission [24].

Our study has some limitations; one is the small sample size which was collected only from one center. Regarding this limitation, concerns about subgroup analysis of this study may increase and further studies are needed to be conducted on a higher number of patients from a larger community of stroke patients which will provide more statistical power and allow for a more accurate estimation of the prevalence and associations between TSH and stroke. Another limitation of our study was the cross‐sectional design which prevents us from identifying causal relationships and future longitudinal studies can help find such correlations.

## 5. Conclusions

In conclusion, although our study did not find a significant relationship between TSH levels and stroke occurrence, it did reveal a notable association between elevated TSH levels and reduced stroke severity at both admission and discharge. This suggests that thyroid function, particularly TSH levels, may influence early neurological outcomes in stroke patients. Given the limitations of our study, especially its small sample size and single‐center design, future multicenter investigations with larger, more diverse cohorts are essential. These should be aimed at exploring the potential of thyroid markers as prognostic tools or therapeutic targets in stroke management.

**Nomenclature**
TSH: thyroid‐stimulating hormone
CVT: cerebral venous thrombosis
AF: atrial fibrillation
TIA: transient ischemic attack
OCP: oral contraceptive pill
DVT: deep vein thrombosis
mRS: modified Rankin score

## Ethics Statement

This study was approved by the Regional Committee of Research Ethics at Tabriz University of Medical Sciences (Ethics Code IR.TBZMED.REC.1402.737). Written informed consent was obtained from all participants or their legally authorized representatives prior to enrollment. All procedures were conducted in accordance with the Declaration of Helsinki [25].

## Conflicts of Interest

The authors declare no conflicts of interest.

## Author Contributions

A.M.: writing—original draft, formal analysis, investigation. A.E.: formal analysis, writing—original draft, investigation. V.S.: conceptualization, methodology, visualization, writing—review and editing, data curation, project administration. A.G.: conceptualization, methodology, writing—review and editing, resources, project administration. K.B.: conceptualization, methodology, writing—original draft, resources, formal analysis, project administration. S.A.A.: writing—original draft, resources, project administration. S.A.: conceptualization, writing—review and editing, resources. S.S.V.: conceptualization, methodology, visualization, supervision, writing—review and editing, project administration. A.M. and A.E. contribute equally and both of them should be considered co‐first authors.

## Funding

The study is supported by Tabriz University of Medical Sciences, 10.13039/501100004366 (70074).

## Data Availability

The data that support the findings of this study are available on request from the corresponding author. The data are not publicly available due to privacy or ethical restrictions.
